# Global guidance for the recognition, diagnosis, and management of tumor‐induced osteomalacia

**DOI:** 10.1111/joim.13593

**Published:** 2022-12-13

**Authors:** Suzanne M. Jan de Beur, Salvatore Minisola, Wei‐bo Xia, Bo Abrahamsen, Jean‐Jacques Body, Maria Luisa Brandi, Roderick Clifton‐Bligh, Michael Collins, Pablo Florenzano, Pascal Houillier, Yasuo Imanishi, Erik A. Imel, Aliya A. Khan, M. Carola Zillikens, Seiji Fukumoto

**Affiliations:** ^1^ Division of Endocrinology and Metabolism, Department of Medicine Johns Hopkins University School of Medicine Baltimore Maryland USA; ^2^ Department of Clinical, Internal, Anesthesiology and Cardiovascular Sciences Sapienza University Rome Italy; ^3^ Department of Endocrinology Key Laboratory of Endocrinology State Key Laboratory of Complex Severe and Rare Diseases NHC Peking Union Medical College Hospital Chinese Academy of Medical Science and Peking Union Medical College Beijing China; ^4^ Open Patient data Explorative Network (OPEN) Department of Clinical Research University of Southern Denmark and Odense University Hospital Odense Denmark; ^5^ Department of Medicine Holbæk Hospital Holbæk Denmark; ^6^ Nuffield Department of Orthopaedics Rheumatology and Musculoskeletal Sciences University of Oxford Oxford UK; ^7^ Department of Medicine CHU Brugmann Université Libre de Bruxelles Brussels Belgium; ^8^ Department of Surgery and Translational Medicine University of Florence University Hospital of Florence Florence Italy; ^9^ Department of Endocrinology and Diabetes Royal North Shore Hospital Sydney Australia; ^10^ Cancer Genetics Unit Kolling Institute Sydney Australia; ^11^ Sydney Medical School University of Sydney Sydney Australia; ^12^ Skeletal Diseases and Mineral Homeostasis Section National Institute of Dental and Craniofacial Research National Institutes of Health Bethesda Maryland USA; ^13^ Centro Traslacional de Endocrinología UC (CETREN‐UC) Endocrinology Department School of Medicine Pontificia Universidad Católica de Chile Santiago Chile; ^14^ Centre de Recherche des Cordeliers INSERM Sorbonne Université Université de Paris Département des Maladies Rénales et Métaboliques Hôpital Européen Georges‐Pompidou Assistance Publique‐Hôpitaux de Paris Paris France; ^15^ Department of Metabolism, Endocrinology, and Molecular Medicine Osaka Metropolitan University Graduate School of Medicine Osaka Japan; ^16^ Division of Endocrinology Indiana University School of Medicine Indianapolis Indiana USA; ^17^ Division of Endocrinology and Metabolism Calcium Disorders Clinic Department of Medicine McMaster University Hamilton Ontario Canada; ^18^ Department of Internal Medicine Erasmus University Medical Center Rotterdam The Netherlands; ^19^ Fujii Memorial Institute of Medical Sciences, Institute of Advanced Medical Sciences Tokushima University Tokushima Japan

**Keywords:** consensus, diagnostic tests/routine, fibroblast growth factor 23, hypophosphatemia, referral and consultation, tumor‐induced osteomalacia

## Abstract

Tumor‐induced osteomalacia (TIO) is a rare paraneoplastic syndrome caused by mesenchymal tumors that secrete fibroblast growth factor 23 (FGF23). Patients present with progressive bone pain, muscle weakness, and fragility fractures. TIO is characterized by hypophosphatemia, excess renal phosphate excretion, and low/inappropriately normal 1,25‐dihydroxyvitamin D (1,25(OH)_2_D) levels. Rarity and enigmatic clinical presentation of TIO contribute to limited awareness among the medical community. Accordingly, appropriate diagnostic tests may not be requested, leading to delayed diagnosis and poorer patient outcomes. We have developed a global guidance document to improve the knowledge of TIO in the medical community, enabling the recognition of patients with TIO and appropriate referral. We provide recommendations aiding diagnosis, referral, and treatment, helping promote a global standard of patient management. We reviewed the literature and conducted a three‐round Delphi survey of TIO experts. Statements were drafted based on published evidence and expert opinions (≥70% consensus required for final recommendations). Serum phosphate should be measured in patients presenting with chronic muscle pain or weakness, fragility fractures, or bone pain. Physical examination should establish features of myopathy and identify masses that could be causative tumors. Priority laboratory evaluations should include urine/serum phosphate and creatinine to assess renal tubular reabsorption of phosphate and TmP/GFR, alkaline phosphatase, parathyroid hormone, 25‐hydroxyvitamin D, 1,25(OH)_2_D, and FGF23. Patients with the clinical/biochemical suspicion of TIO should be referred to a specialist for diagnosis confirmation, and functional imaging should be used to localize causative tumor(s). Recommended treatment is tumor resection or, with unresectable/unidentifiable tumors, phosphate salts plus active vitamin D, or burosumab.

## Graphical abstract

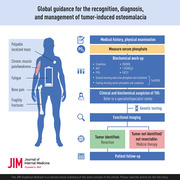

**Abbreviations**
%TRP: the fraction (or percentage) of filtered phosphorus that is reabsorbed by renal tubules
1,25(OH)_2_D: 1,25‐dihydroxyvitamin D
25(OH)D: 25‐hydroxyvitamin D
ADHR: autosomal dominant hypophosphatemic rickets
ALP: alkaline phosphatase
ARHR: autosomal recessive hypophosphatemic rickets
CSHS: cutaneous skeletal hypophosphatemia syndrome
CT: computed tomography
FDG: fluorodeoxyglucose
FGF: fibroblast growth factor
HHRH: hereditary hypophosphatemic rickets with hypercalciuria
HBS: hungry bone syndrome
IIH: idiopathic infantile hypercalcemia
MRI: magnetic resonance imaging
NF1: neurofibromatosis type 1
NPT2: type II sodium phosphate cotransporter
PET: positron emission tomography
PMT: phosphaturic mesenchymal tumor
PTH: parathyroid hormone
QoL: quality of life
SPECT: single‐photon@@@ emission computed tomography
TIO: tumor‐induced osteomalacia
TmP/GFR: ratio of tubular maximum reabsorption of phosphate to glomerular filtration rate
TRP: tubular reabsorption of phosphate
XLH: X‐linked hypophosphatemia

## Introduction

**Fig. 1:**
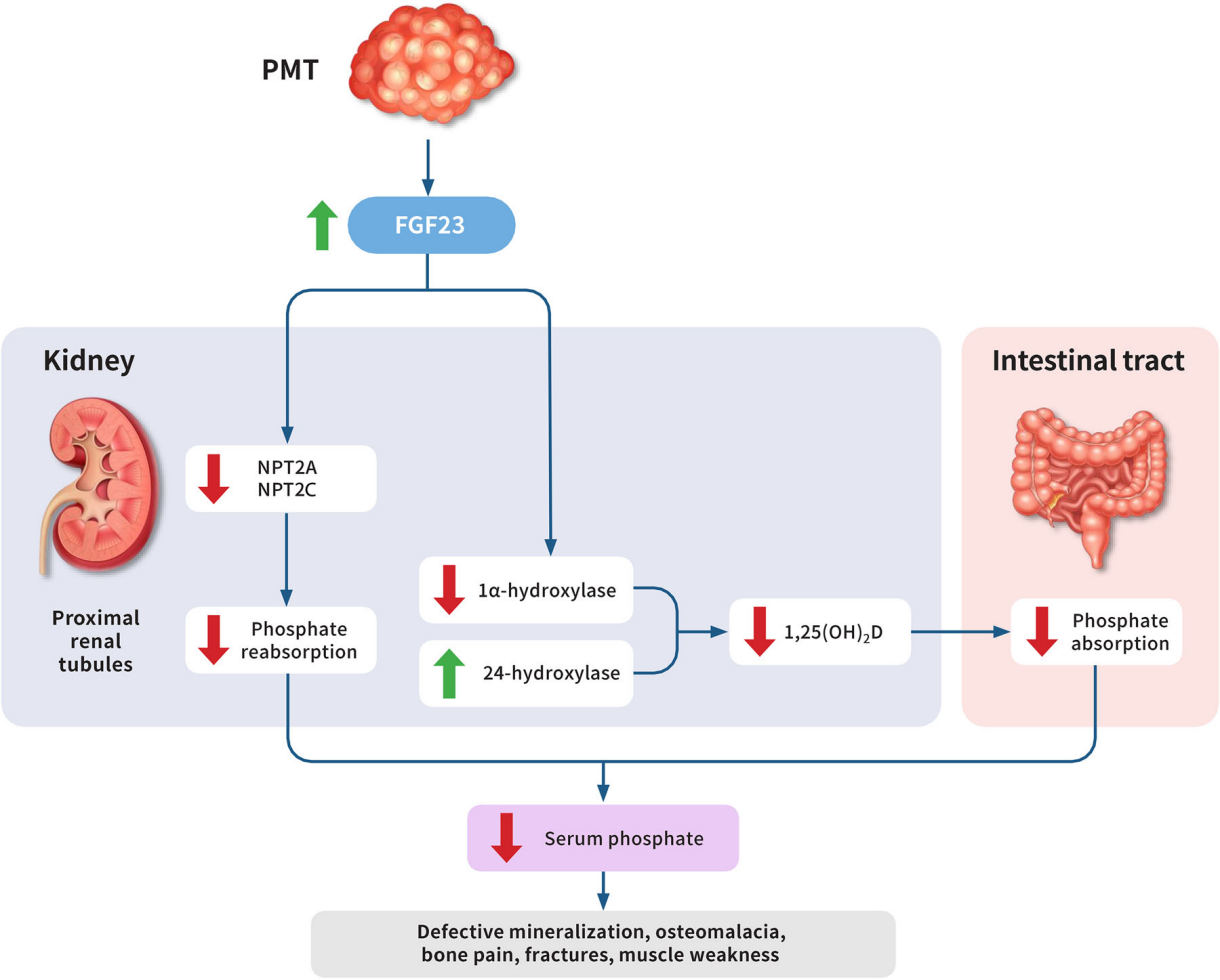
Pathophysiology of tumor‐induced osteomalacia (TIO). 1,25(OH)_2_D, 1,25‐dihydroxyvitamin D; FGF23, fibroblast growth factor 23; NPT2, type II sodium phosphate cotransporter; PMT, phosphaturic mesenchymal tumor. Source: Figure adapted from Minisola et al., Courbebaisse et al., and Saraff et al. [3, 5, 6].

Tumor‐induced osteomalacia (TIO) is a rare paraneoplastic syndrome, resulting from the secretion of the phosphaturic hormone fibroblast growth factor 23 (FGF23) from a morphologically and genetically distinct tumor of soft tissue and bone, termed a phosphaturic mesenchymal tumor (PMT) (Fig. 1) [1, 2, 3]. The tumors are typically benign, but malignant tumors can occur [3, 4]. Patients usually present with a nonspecific constellation of symptoms, including diffuse bone pain, fractures, fatigue, and muscle weakness, which can make TIO difficult to recognize. In the very rare cases of TIO in children, patients may present with gait disturbances, growth retardation, and skeletal deformities.

The hallmark biochemical feature of TIO, hypophosphatemia, often goes undetected because serum phosphate is not routinely measured [7] or may be erroneously attributed to another cause (Table 1). Furthermore, the full biochemical evaluations necessary to make the diagnosis of TIO—decreased renal tubular reabsorption of phosphate (TRP), inappropriately normal or low 1,25‐dihydroxyvitamin D (1,25[OH]_2_D), and increased (or inappropriately normal) circulating FGF23—are specialized tests that are unfamiliar to many clinicians.

**Table 1:** Differential diagnosis of hypophosphatemia^*a*^

Causes of hypophosphatemia
1. Hypophosphatemia mainly caused by excessive renal excretion
1.1. FGF23‐mediated
1.1.1. Genetic
ADHR (*FGF23*)
ARHR (*DMP1*, *ENPP1*, *FAM20C*) [8]
CSHS/linear sebaceous nevus syndrome (*RAS*: *KRAS/HRAS*, *NRAS*) [9, 10]
Fibrous dysplasia of bone (*GNAS*)
Jansen's metaphyseal chondrodysplasia (*PTH1R*) [11]
NF1 (*NF1*) [12, 13]
Osteoglophonic dysplasia (*FGFR1*) [8]
XLH (*PHEX*)
1.1.2. Acquired
Chronic alcohol consumption [14]
Iron polymaltose, carboxymaltose or saccharated ferric oxide infusions
TIO
1.2. Non–FGF23‐mediated
HHRH (*SLC34A3*) [15]
Hyperparathyroidism
IIH (*SLC34A1*) [16]
Renal Fanconi syndrome^b^
2. Impaired actions of vitamin D metabolites
Vitamin D deficiency
Vitamin D metabolism defects
3. Malabsorption, malnutrition
Low dietary intake
Impaired dietary absorption (e.g., celiac disease, gastric bypass, inflammatory bowel disease)
Phosphate binders (sevelamer, antacids containing calcium, magnesium, aluminum)
Alcoholism
Premature infants
4. Transcellular shifts
Diabetic ketoacidosis [17]
Hyperventilation
Refeeding syndrome
Respiratory alkalosis
5. Drugs not included in 1.1
Aminoglycosides
Antiretrovirals (tenofovir, adefovir)
Bisphosphonates
Catecholamines
Chemotherapies (cisplatin, ifosfamide, streptozocin)
Diuretics (acetazolamide, thiazides, loop diuretics)
Glucose or insulin infusion
Imatinib
Mannitol
Salicylate
Sirolimus (rapamycin)
Tetracyclines

Abbreviations: ADHR, autosomal dominant hypophosphatemic rickets; ARHR, autosomal recessive hypophosphatemic rickets; CSHS, cutaneous skeletal hypophosphatemia syndrome; *DMP1*, dentin matrix acidic phosphoprotein 1; *ENPP1*, ectonucleotide pyrophosphatase/phosphodiesterase 1; *FAM20C*, family with sequence similarity 20, member C; FGF23, fibroblast growth factor 23; *FGFR1*, fibroblast growth factor receptor 1; *GNAS*, guanine nucleotide binding protein, alpha stimulating; HHRH, hereditary hypophosphatemic rickets with hypercalciuria; *HRAS*, HRas proto‐oncogene, GTPase; IIH, idiopathic infantile hypercalcemia; *KRAS*, KRas proto‐oncogene; NF1, neurofibromatosis type 1; *NF1*, neurofibromin 1; *NRAS*, NRAS proto‐oncogene, GTPase; *PHEX*, phosphate regulating endopeptidase, X‐linked; *RAS*, rat sarcoma virus; *SLC34A1*, solute carrier family 34 member 1; *SLC34A3*, solute carrier family 34 member 3; TIO, tumor‐induced osteomalacia; XLH, X‐linked hypophosphatemia.

^a^ Adapted from Clarke et al and Imel et al. [18, 19].

^b^ Osteomalacia associated with adult acquired Fanconi's syndrome is thought to result from hypophosphatemia and relative calcitriol deficiency.

There are many challenges associated with the diagnosis of TIO. As a rare disease, the clinical presentation is currently poorly recognized, and because serum phosphate is not routinely included in standard chemistry panels [1, 7], an increased awareness of the importance of specifically requesting its measurement is required [3, 20, 21, 22]. There is also often a failure to understand the association between low serum phosphate and presenting clinical symptoms [3, 20, 21, 22]. Furthermore, the lengthy differential diagnosis of hypophosphatemia, coupled with a complicated diagnostic evaluation [3, 20, 21, 22], can also impede diagnosis, as can confounding genetic causes that mimic the biochemical and clinical presentation of TIO [3, 20, 21, 22]. Finally, the localization of tumors can also be challenging [3, 20, 21, 22, 23]. These obstacles often culminate in a significant delay in the diagnosis of TIO, and patients can remain untreated for years. A delay of 2.5–3.5 years has recently been reported between initial presentation and tumor‐related treatment, with a delay of >2 years in 80% of cases [7, 24]. This can lead to progressive and severe disabilities, loss of mobility, severe bone pain, fractures, bone deformity, muscle weakness, and decreased quality of life (QoL) [1, 3].

TIO is curable if the causative tumor can be completely resected, which is the definitive and preferred treatment [3, 25, 26]. When the tumor is not located, the resection of a primary or recurrent tumor is unsuccessful or not possible, or the tumor is metastatic, medical therapy is required to improve symptoms and diminish skeletal complications [3, 22, 26].

Although TIO is increasingly recognized, its global incidence and prevalence are unknown. Fewer than 1000 cases of TIO have been reported in the literature [1]. A singular epidemiological study estimated the prevalence of TIO to be 0.70 per 100,000 persons (0.43 per 100,000 adults) [27]. Actual incidence and prevalence rates could be higher due to underdiagnosis and underreporting. No sex or ethnic differences have been observed [28].

Patients with TIO can be seen initially by a variety of health‐care providers, including general practitioners, orthopedists, neurologists, rheumatologists, endocrinologists, nephrologists, and physical medicine/rehabilitation specialists. Clinical practice guidance for rare diseases can support clinicians in identifying, diagnosing, and treating patients and thus improve patient care and outcomes [29]. Several recently published expert reviews describe TIO and its management [1, 20, 21, 22]; however, there remains a critical need for practical clinical guidance to assist general medical practitioners in recognizing the presenting symptoms of TIO and understanding the subsequent diagnostic pathway, which will facilitate the rapid referral of patients with TIO to specialist clinicians for treatment.

## Methods

### Writing committee

The author group is made up of peer group– and scientific society–recommended experts representing five continents, comprising a wide range of specialists, recommended based on their publication records and the number of patients with TIO they have managed.

### Literature search

Due to the rarity of TIO, there is a lack of high‐quality evidence to be graded (such as randomized controlled trials) for this disease. Therefore, the authors decided to provide recommendations and guidance based on expert opinions and the Delphi method rather than using the Grading of Recommendations, Assessment, Development and Evaluations (GRADE) approach. The PubMed database was searched up to August 2022, using the following search terms: “tumor‐induced osteomalacia,” OR “tumour‐induced osteomalacia,” OR “oncogenic osteomalacia.” Further articles were searched in PubMed when additional information was required. The search retrieved more than 820 results, with 83 of those articles referenced here.

### Delphi method

Recommendation statements were determined using the Delphi method. The voting group included the full author list (15 authors) plus one additional expert invited by the author group. Authors were asked to provide a level of agreement on recommendation statements using a five‐point scale (strongly disagree, disagree, neither agree/disagree, agree, or strongly agree) via an online survey platform, anonymous from each other. When a ≥70% consensus level was not reached, recommendations were modified based on author suggestions and reviewed again by the voting panel. The process was repeated over three rounds of voting until a consensus level of ≥70% was achieved. Recommendations were graded as strong when a consensus of “strongly agree” was reached and moderate when a consensus of “agree” was reached.

## The importance of early recognition and diagnosis of TIO

TIO has an initial misdiagnosis rate of 95.1% [30]. Misdiagnosis can result in no treatment, or the wrong treatment being administered [31, 32]. Patients with TIO are misdiagnosed with a variety of rheumatological or musculoskeletal disorders, including lumbar disc herniation, spondyloarthritis, osteoporosis, rheumatoid arthritis, bone metastases, and connective tissue diseases [1, 30]. Patients are sometimes diagnosed with neurological and muscular disorders, including motor neuron disease, multiple sclerosis, polymyalgia rheumatica, myositis, stroke, or functional somatization [30] (authors’ personal experiences). As parathyroid hormone (PTH) can be elevated because of FGF23 excess in patients with TIO, primary hyperparathyroidism is also a common misdiagnosis. In some cases, TIO goes unrecognized because presenting symptoms are attributed to coexisting medical conditions [30]. Early recognition and diagnosis of TIO can prevent disease progression, reverse damage already incurred by excess FGF23, reduce the risk of permanent deformity, and avert harm from misdiagnosis and inappropriate or lack of treatment. It should be noted that TIO does not have an *International Classification of Diseases‐10* code. This is a barrier to monitoring management in health databases and designating the accurate diagnosis in electronic patient records.

## Recognizing clinical signs and symptoms that raise suspicion for TIO

**Fig. 2:**
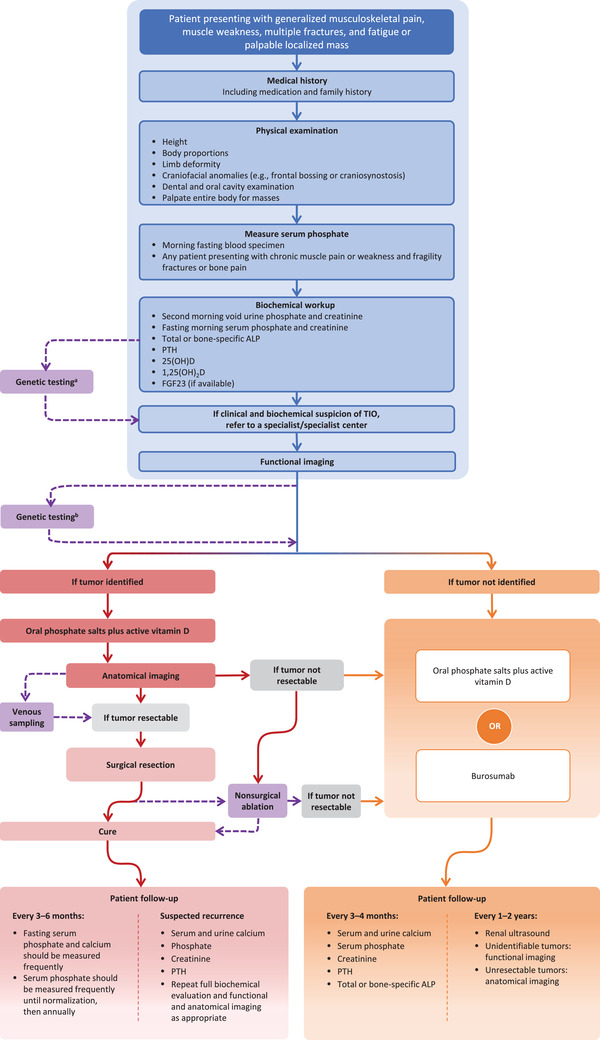
Tumor‐induced osteomalacia (TIO) diagnostic and treatment algorithm. 1,25(OH)_2_D, 1,25‐dihydroxyvitamin D; 25(OH)D, 25‐hydroxyvitamin D; ALP, alkaline phosphatase; FGF23, fibroblast growth factor 23; PTH, parathyroid hormone. ^a^Perform genetic testing before imaging in children, young adults, or those with suggestive family history. ^b^Perform genetic testing in adults without family history and negative functional imaging.

Initial presenting signs and symptoms of TIO are often nonspecific and develop gradually. Rarely, patients present with a palpable localized mass [30]. A directed medical and familial history can aid differential diagnosis, highlight clinical red flags, and enable diagnosis by exclusion (Table 2, Fig. 2).

**Table 2:** Expert recommendations: (A) strong recommendation, (B) moderate recommendation

Recommendations	
Recognition and referral	Measure serum phosphate in any patient presenting with chronic muscle pain, weakness, fragility fractures, or bone pain. **(A)** Perform genetic testing for hereditary hypophosphatemic disorders in a patient who has a family history of hypophosphatemia, personal or family history of short stature, lower limb deformity, or extensive dental anomalies. **(B)** Consider genetic testing to exclude hereditary hypophosphatemic disorders in a patient who has hypophosphatemia, renal phosphate wasting, elevated or inappropriately normal FGF23 with no evidence of personal or family history that may suggest a genetic cause, when no tumor can be identified with appropriate imaging, or when onset of disease is in childhood or young adulthood. **(B)** Perform a thorough physical examination in a patient suspected of having TIO, including height measurement, assessment of body proportions, limb deformity, craniofacial anomalies, including frontal bossing or craniosynostosis, and dental and oral cavity examination. Palpate the entire body for evidence of masses. **(A)** In patients with an unclear diagnosis, perform priority laboratory evaluations (indicators of TIO), including: ◦ Second morning void urine phosphate and creatinine, plus fasting morning serum phosphate and creatinine, for calculation of TmP/GFR^a^ ◦ Total or bone‐specific ALP ◦ Intact PTH ◦ 25(OH)D ◦ 1,25(OH)_2_D (when available). **(B)** Where clinically available, measure circulating FGF23^b^ in those with confirmed hypophosphatemia, renal phosphate wasting (by TmP/GFR), and low or inappropriately normal 1,25(OH)_2_D. **(A)** After excluding hereditary and acquired causes of renal hypophosphatemia, suspect TIO in patients with the following pattern of laboratory assessments: ◦ Serum phosphate ↓ ◦ TmP/GFR ↓ ◦ Serum calcium normal/slightly ↓ ◦ 25(OH)D^c^ normal/↓ ◦ 1,25(OH)_2_D inappropriately normal/↓ ◦ ALP ↑/↑↑ ◦ PTH normal/↑ ◦ Intact FGF23^d^ ↑/inappropriately normal ◦ C‐terminal FGF23^d^ ↑/inappropriately normal. **(B)** Refer patients with clinical and biochemical suspicion of TIO to a specialist/specialist center experienced in the treatment of rare metabolic bone disease for confirmation of diagnosis. **(A)**
Imaging and tumor localization	Once the diagnosis of TIO is biochemically established, perform functional imaging. Follow with focused anatomical imaging using the most appropriate modality (MRI, CT) based on the suspected tumor location present on functional imaging. **(A)** Employ ^68^Ga‐based or ^64^Cu‐based PET/CT (^68^Ga/^64^Cu‐DOTATATE, ‐DOTANOC, or ‐DOTATOC) as first‐line functional imaging. When unavailable, substitute octreotide scan (^99m^Tc‐ or ^111^In‐pentetreotide scan) or FDG‐PET as alternatives, in this order.^e^ **(B)** Consider venous sampling of FGF23 levels for differentiating between two possible lesions seen on functional imaging to confirm (or to help localize) a suspected causative tumor. **(B)**
Treatment and follow‐up	Initiate medical therapy with oral phosphate salts plus active vitamin D promptly in patients when TIO is biochemically confirmed in parallel with diagnostic imaging and in preparation for surgical resection of the culprit tumor. **(A)** Completely resect the culprit tumor (with tumor‐free margins) for definitive first‐line TIO treatment to achieve cure. **(A)** In patients with confirmed TIO and with unresectable or unidentifiable tumors, treat with oral phosphate salts plus active vitamin D, or burosumab, considering treatment availability, clinical response, and adverse events. **(B)** Follow patients with TIO and unresectable or unidentifiable tumors on stable doses of oral phosphate salts plus active vitamin D, or burosumab, every 3–4 months with tests for serum and urine calcium, phosphate, creatinine, PTH, total or bone‐specific ALP, and renal ultrasound.^f^ More frequent monitoring is required when initiating treatment and after dose adjustment. **(B)** Follow patients with TIO and unidentifiable tumors with functional imaging and those with unresectable tumors with anatomical imaging, every 1–2 years. **(A)**

*Note*: Recommendations were graded as strong when a consensus of “strongly agree” was reached and moderate when a consensus of “agree” was reached.

Abbreviations: 1,25(OH)_2_D, 1,25‐dihydroxyvitamin D; 25(OH)D, 25‐hydroxyvitamin D; ALP, alkaline phosphatase; CT, computed tomography; FDG, fluorodeoxyglucose; FGF23, fibroblast growth factor 23; MRI, magnetic resonance imaging; PET, positron emission tomography; PTH, parathyroid hormone; TIO, tumor‐induced osteomalacia; TmP/GFR, ratio of tubular maximum reabsorption of phosphate to glomerular filtration rate.

^a^ Calculation of TmP/GFR only when hypophosphatemia is confirmed.

^b^ Intact FGF23 preferred.

^c^ Vitamin D deficiency/insufficiency is frequent in the general population and 25(OH)D level can be low concurrently with TIO.

^d^ Give preference to measurement of intact FGF23.

^e^ Dependent on local availability of imaging modalities.

^f^ Use renal ultrasound/other radiological tools if necessary to detect nephrocalcinosis and nephrolithiasis annually; monitor 24‐h urinary calcium to identify the need for adjusting treatment before nephrocalcinosis becomes visibly present.

### Medical history

A careful medical history should include the age of onset and accompanying symptoms, which may include bone pain, muscle weakness and pain, fatigue, decreased exercise tolerance, gait disturbance, height loss, increased thoracic kyphosis, pectus carinatum, and fractures [30, 33, 34, 35, 36, 37]. Cases have been reported in individuals as young as 9 months old [38], with a median age of onset of 46 years and a range of 9 months–90 years [7, 39]. Previous accounts indicated that equal proportions of men and women are affected by TIO [35]; however, more recent evidence from systematic reviews suggests that more men than women are diagnosed [7, 40]. A systematic review of 895 TIO cases reported that, of the 858 cases with information on sex, 58.3% were male and 41.7% were female [7]. Similar results were reported in another recent systematic review [40]. Bone pain in the distal extremities is the most common initial symptom in adults [37], with ankles and feet being the most often affected. Other frequently reported sites of pain include the upper and lower back and neck, sternum, and shoulders, as well as diffuse/generalized bone pain [30]. Fractures commonly occur in the ribs, vertebral bodies, femoral neck or shaft, and pelvis [30, 33, 35]. In line with this, evidence also suggests that patients with TIO have notably lower bone mineral density and strength [24] compared with not only healthy controls [41, 42] but also patients with X‐linked hypophosphatemia (XLH) [41]. In the rare cases in children, there may be delayed motor milestones, lower extremity deformity, failure to ambulate, and other rachitic symptoms [43, 44] (authors’ clinical observations).

Medication history should be carefully assessed for iatrogenic causes of hypophosphatemia (Table 1) [45, 46, 47].

### Family history

TIO should be suspected in patients with suggestive symptoms and clinical presentation [22, 48] without evidence of personal or family history of hereditary hypophosphatemia. Taking an accurate family history is particularly important in children because genetic causes of hypophosphatemia are much more common than TIO. Ideally, all children and young adults suspected of having TIO should undergo genetic testing to exclude genetic causes of hypophosphatemia. Because the onset of heritable causes can sometimes be subtle, not becoming apparent until adulthood, genetic testing should be strongly considered in adults with chronic hypophosphatemia due to renal phosphate wasting for whom the diagnosis of TIO is being considered, especially when the tumor cannot be identified.

## Evaluation of patients with suspected TIO

Diagnosis of TIO depends on a combination of accurate biochemical evaluation, medical and family history (see the “Recognizing clinical signs and symptoms that raise suspicion for TIO” section), physical examination, and tumor identification (Fig. 2). Biochemical tests are necessary but not sufficient for the final diagnosis of TIO, and differential diagnosis from other FGF23‐related renal phosphate wasting disorders is needed [1, 3].

The potential for the biochemical differentiation of TIO with XLH continues to be investigated. One retrospective analysis indicated that patients with TIO had significantly higher levels of FGF23 and alkaline phosphate, significantly lower levels of phosphorus and 1,25(OH)_2_D, but similar levels of 25‐hydroxyvitamin D (25[OH]D) compared with patients with XLH [36]. A potential role for sclerostin in the differentiation of TIO and XLH has also been proposed, with one cross‐sectional study reporting decreased levels in patients with TIO and increased levels in patients with XLH [49]. Other interesting approaches being explored for the identification of patients with TIO include creating a metabolome profile, which has the potential to assist in determining potential biomarkers of TIO in the future [50].

### Physical examination

An initial physical examination should investigate the main symptoms of TIO (see the “Recognizing clinical signs and symptoms that raise suspicion for TIO” section), including the examination of commonly affected musculoskeletal sites, skin, and oral cavity. The entire body should be palpated for masses [35], which are often very small. Patients may have tenderness to palpation over the tibia or sites of suspected fracture due to osteomalacia [33]. The following should be examined: (1) height, to ascertain whether the patient is shorter than mean predicted parental height consistent with genetic cause, and to establish height loss; (2) lower limbs, for deformities that may alert to a genetic cause in adults; (3) teeth, for numerous fillings, missing teeth, dentures, or implants that may be indicative of genetic causes; (4) head, for frontal bossing or craniosynostosis indicating genetic causes; (5) hearing, for hearing loss, which may suggest a genetic cause; however, note that hearing loss is common in the general population; (6) muscle strength, for proximal muscle weakness and mobility; (7) spine, for increased thoracic kyphosis and pain to palpation, indicating occult vertebral fractures; and (8) gait for the antalgic or waddling feature that can be seen in osteomalacia.

### Biochemical workup and features

The challenges associated with diagnostic testing have been recently reviewed by Brandi et al. [20]; for the purposes of this global guidance document, we will focus on the most salient points for clinicians to bear in mind. The first necessary step to diagnosis requires measuring serum phosphate in anyone being evaluated for musculoskeletal symptoms (Fig. 2). Serum phosphate should be measured on a morning, fasting blood specimen [1]. Serum phosphate concentration is normally higher in young children and decreases with age [51]; therefore, it is important to make sure that an age‐specific normal range is used. In adults, hypophosphatemia is defined as levels below 0.8 mmol/L (2.5 mg/dL) [52]. To establish chronic hypophosphatemia, there must be two values below the normal range on at least two occasions ≥2 weeks apart.

One study showed that, among 144 patients with TIO, 43.1% of cases with hypophosphatemia were not treated or misdiagnosed, and only 11.8% of patients seeking medical care for the first time were tested for serum phosphate [30].

Comprehensive laboratory evaluation should include complete blood count, fasting serum phosphate, serum creatinine, calcium, albumin, and total alkaline phosphatase (ALP). Additional variables, including PTH, 25(OH)D, 1,25(OH)_2_D, and FGF23, should also be measured (Table 3, Fig. 2) [1, 7, 34]. It should be emphasized that not all assays are standardized, and not all labs use the same reference values, so care must be taken when interpreting results.

Measurement of 25(OH)D levels is necessary for diagnosing vitamin D deficiency, a common cause of hypophosphatemia and rickets/osteomalacia [1, 34] that must be differentiated from TIO. However, vitamin D deficiency/insufficiency is frequent in the general population, and a low 25(OH)D level does not rule out the possibility of concurrent TIO. In fact, persistent hypophosphatemia after correction of vitamin D deficiency should prompt consideration that the patient may have TIO. Iron deficiency may indicate a need for potentially different patient management.

### Serum phosphate and ratio of tubular maximum reabsorption of phosphate to glomerular filtration rate (TmP/GFR)

Three primary mechanisms of hypophosphatemia exist: increased renal excretion (due to decreased renal tubular reabsorption), decreased intake/intestinal absorption, and shifts from the extracellular to intracellular compartments or bone (Fig. 1). Once chronic hypophosphatemia is established, the renal phosphate excretion should be determined by calculating TRP and then deriving ratio of tubular maximum reabsorption of phosphate to glomerular filtration rate (TmP/GFR). Increased TmP/GFR is consistent with impaired intestinal absorption or intake; TmP/GFR is reduced in conditions of renal phosphate losses, including but not limited to TIO. Simultaneous fasting serum phosphate and creatinine, and urine phosphate and creatinine (second morning void) should be collected to assess renal phosphate reabsorption [3]. TRP—or %TRP, the fraction (or percentage) of filtered phosphorus that is reabsorbed by renal tubules—can be used by itself to provide a convenient estimate of renal phosphate handling [3]. %TRP is also used in the determination of TmP/GFR, which is a more accurate assessment of renal phosphate reabsorption. %TRP may be calculated using the following equation [1]:

$$100\;\times \;\left(1\;-\;\frac{\mathrm{urine}\;\mathrm{phosphate}\;\times \;\mathrm{serum}\;\mathrm{creatinine}}{\mathrm{serum}\;\mathrm{phosphate}\;\times \;\mathrm{urine}\;\mathrm{creatinine}}\right)$$

TmP/GFR can be derived using the Walton–Bijvoet nomogram [53] or the following equations [54]:

$$\begin{array}{c} \mathrm{For}\;\mathrm{TRP}\leq 0.86\;(86\%)\;:\\ \;\mathrm{TmP}/\mathrm{GFR}=\mathrm{TRP}\times \mathrm{phosphate}\;\mathrm{For}\;\mathrm{TRP}>0.86\left(86\%\right)\;:\\ \;\mathrm{TmP}/\mathrm{GFR}=\left(0.3\times \mathrm{TRP}\right)/\left[1-\left(0.8\times \mathrm{TRP}\right)\right]\\ \;\times \;\mathrm{phosphate} \end{array}$$

TmP/GFR and %TRP can also be calculated using many available online programs [1]. In children, the equation used by Stark et al. may be more accurate [55].

#### Low TmP/GFR

Hypophosphatemia with low TmP/GFR and/or %TRP should raise suspicion for TIO [1, 7, 28]. However, hypophosphatemia with increased renal excretion may be FGF23‐mediated or non‐FGF23‐mediated (Table 1). Measuring 1,25(OH)_2_D alongside FGF23 differentiates between FGF23‐mediated and non‐FGF23‐mediated disorders. 1,25(OH)_2_D is typically low or inappropriately normal in patients with TIO [1, 7] because FGF23 reduced its synthesis and increases its catabolism. FGF23 should be interpreted with caution, as not all patients with TIO have elevated FGF23 [30, 39, 56], and elevated FGF23 can also be found in other diseases [3, 57]. Additionally, FGF23 assays are not standardized and can provide variable results for intact FGF23 measurement [58]. FGF23 levels can also be affected by medications (Table 1), decreased renal function (even small decreases in GFR), and high‐phosphate diets [59, 60]. Access to FGF23 testing varies globally: There is geographic variability in which assays are available (if any) and in reimbursement for its clinical use. When FGF23 testing is required, local specialists and/or pathology laboratories should be consulted about the appropriate procedures. If intact or C‐terminal FGF23 is low or low normal, the clinician should consider diagnoses other than TIO.

### Referral and clinical specialties

Once the presence of chronic hypophosphatemia due to excess renal phosphate excretion and TIO is suspected, patients should be referred to an appropriate specialist (such as a metabolic bone specialist or endocrinologist) at secondary or tertiary centers with experience in treating TIO for diagnosis confirmation (Table 2, Fig. 2).

### Genetic testing

It is important to rule out hereditary hypophosphatemia before making the diagnosis of TIO. Lack of family history does not entirely preclude heritable forms of hypophosphatemia, as de novo mutations are common [28, 61]. Furthermore, genetic testing may be useful in patients suspected of having TIO, because some genetic forms of hypophosphatemia may present in adulthood, such as autosomal dominant hypophosphatemic rickets [62, 63], or be unrecognized until adulthood [8]. Once FGF23 excess is established and/or genetic causes and other causes of hypophosphatemia have been ruled out in children, young adults, or those with suggestive family history, imaging to locate the causative tumor is performed. Genetic testing should also be considered in adults with negative functional imaging results (Table 4, Fig. 2).

**Table 3:** Biochemical features of tumor‐induced osteomalacia (TIO) and reference ranges (in adults)

Parameter	Adult reference range^a^	Feature in TIO
Serum phosphate^b^	0.81–1.45 mmol/L (2.51–4.49 mg/dL)	Decreased
Serum ALP	Male: 45–125 U/L Female: 35–100 U/L	Increased
Serum calcium	2.15–2.55 mmol/L (8.6–10.2 mg/dL)	Slightly decreased/normal
Intact FGF23^c^	0.45–1.86 pmol/L^d^ (11.7–48.6 pg/mL)	Increased/inappropriately normal
C‐terminal FGF23^c^	21.6–91.0 RU/mL	Increased
Intact PTH	1.27–6.9 pmol/L (12.0–65.0 pg/mL)	Increased/normal
1,25(OH)_2_D	47–130.3 pmol/L (19.6–54.3 pg/mL)	Decreased/inappropriately normal
25(OH)D	75–125 nmol/L (30–50 ng/mL)	Normal^e^
TmP/GFR	0.80–1.35 mmol/L (2.48–4.18 mg/dL)	Decreased
%TRP	85–95	Decreased

Abbreviations: %TRP, the fraction (or percentage) of filtered phosphorus that is reabsorbed by renal tubules; 1,25(OH)_2_D, 1,25‐dihydroxyvitamin D; 25(OH)D, 25‐hydroxyvitamin D; ALP, alkaline phosphatase; FGF23, fibroblast growth factor 23; PTH, parathyroid hormone; RU, reference units; TmP/GFR, ratio of tubular maximum reabsorption of phosphate to glomerular filtration rate.

^a^ Not all assays are standardized, and not all labs use the same reference values. *Values given are adult normal ranges; pediatric normal ranges can differ*. It is important to recognize that the normal levels of serum or plasma phosphate, ALP, and TmP/GFR are all higher in children than in adults. TmP/GFR normal range varies according to age and sex.

^b^ Plasma phosphate is commonly lower than serum phosphate by 0.06–0.1 mM.

^c^ Results are assay‐dependent: four assays are currently available to measure intact FGF23, from Immutopics International, Quidel Corporation, Athens, OH, USA; DiaSorin, Stillwater, MN, USA; Determiner CL FGF23, Minaris Medical, Tokyo; and Kainos Laboratories International, Tokyo, Japan (not commercially available for clinical use); one assay is available to measure C‐terminal FGF23, from Immutopics International, Quidel Corporation, Athens, OH, USA.

^d^ An intact FGF23 level >30 pg/ml is a sensitive cut‐off point for the diagnosis of FGF23‐mediated hypophosphatemia [64].

^e^ Vitamin D deficiency/insufficiency is frequent in the general population, and 25(OH)D levels can be low concurrently with TIO.

**Table 4:** Gene test panel for eliminating genetic causes of hypophosphatemia

Gene	Disorder
*DMP1*	Autosomal recessive hypophosphatemic rickets [8]
*ENPP1*	Autosomal recessive hypophosphatemic rickets [8]
*FAM20C* ^a^	Autosomal recessive hypophosphatemic rickets [8]
*FGF23*	Autosomal dominant hypophosphatemic rickets [8]
*FGFR1*	Osteoglophonic dysplasia [8]
*GNAS*	Fibrous dysplasia/McCune‐Albright syndrome [8]
*NF1*	Neurofibromatosis type 1 [12, 13]
*PHEX*	XLH [8]
*PTH1R*	Jansen's metaphyseal chondrodysplasia [8]
*RAS: HRAS, NRAS*	Cutaneous skeletal hypophosphatemia syndrome [9]
*SLC34A1*	Hypophosphatemia and nephrocalcinosis [16]
*SLC34A3*	Hereditary hypophosphatemic rickets with hypercalciuria [15]

Abbreviations: *DMP1*, dentin matrix acidic phosphoprotein 1; *DMP4*, dentin matrix acidic phosphoprotein 4; *ENPP1*, ectonucleotide pyrophosphatase/phosphodiesterase 1; *FAM20C*, family with sequence similarity 20, member C; *FGF23*, fibroblast growth factor 23; *FGFR1*, fibroblast growth factor receptor 1; *GNAS*, guanine nucleotide binding protein, alpha stimulating; *HRAS*, HRAS proto‐oncogene, GTPase; *NF1*, neurofibromin 1; *NRAS*, NRAS proto‐oncogene, GTPase*; PHEX*, phosphate regulating endopeptidase, X‐linked; *PTH1R*, parathyroid hormone 1 receptor; *RAS*, rat sarcoma virus; *SLC34A1*, solute carrier family 34 member 1; *SLC34A3*, solute carrier family 34 member 3; XLH, X‐linked hypophosphatemia.

^a^ Also referred to as *DMP4* [10].

### Imaging and tumor localization

A systemic approach to tumor localization begins with functional imaging and is followed by anatomic imaging (Table 2, Fig. 2). Functional imaging includes somatostatin receptor imaging (^68^Ga/^64^Cu‐DOTATATE, ‐DOTANOC, or ‐DOTATOC, or octreotide scan) and fluorodeoxyglucose positron emission tomography [65]; whole‐body imaging is typically necessary [31, 35, 66]. See Table 5 for reported sensitivity and specificity of imaging methods. If a suspicious lesion is found with functional imaging, then anatomical imaging is indicated for more precise localization.

**Table 5:** Reported sensitivity and specificity for various imaging methods^*a*^

Method	Study	Reported sensitivity (%)	Reported specificity (%)
^68^Ga‐DOTA‐TATE PET/CT	Clifton‐Bligh et al. [67]	100	
	Breer et al. [68]	100	
	Jadhav et al. [69]	100	
	Zhang et al. [70]	100	90.9
	Agrawal et al. [71]	83.3	
	El‐Maouche et al. [72]	54.5	
	Satyaraddi et al. [73]	100	
	Hou et al. [74]	94.7	
^68^Ga‐DOTA‐NOC PET/CT	Bhavani et al. [75]	90.0	
	He et al. [76]	94.1	75.0
^68^Ga‐DOTA‐TOC PET/CT	Paquet et al. [77]	73.0	66.7
^68^Ga‐DOTA‐JR11 PET/CT	Hou et al. [74]	57.9	
Octreoscan SPECT/CT	Jan de Beur et al. [78]	71.0	
	Jing et al. [79]	86.3	99.1
	Chong et al. [80]	94.7	36.4
	Jadhav et al. [69]	100	
	El‐Maouche et al. [72]	36.3	
^18^FDG‐PET/CT	Jagtap et al. [81]	80.0	
	Chong et al. [80]	88.0	36.0
	Jadhav et al. [69]	50.0	
	Agrawal et al. [71]	83.3	
	El‐Maouche et al. [72]	36.3	86.0
	Jain et al. [82]	87.5	
^99^mTc‐HYNIC‐TOC	Jing et al. [79]	86.3	99.1
	Shi et al. [83]	86.7	
^18^F‐AIF‐NOTA‐Octreotide PET/CT	Long et al. [84]	87.5	100

Abbreviations: CT, computed tomography; FDG, fluorodeoxyglucose; PET, positron emission tomography; SPECT, single‐photon emission computed tomography.

^a^ Adapted from Jiang et al. [85].

To confirm tumor localization and plan for surgery, magnetic resonance imaging and computed tomography are generally recommended due to their high resolution. Radiography and ultrasound may also be used [22]. The most appropriate method depends on the suspected anatomical location [1, 3]. Local availability of imaging techniques influences the detection rate of the tumor.

In some instances, venous sampling of FGF23 levels can be used to localize or confirm a suspected causative tumor or differentiate between two possible lesions seen on functional imaging [58, 86, 87]. Venous sampling is not as useful in localizing tumors when imaging studies do not identify suspicious lesions [88] (Table 2, Fig. 2).

## Management of TIO

### Surgery

When the causative tumor has been identified and localized, complete tumor resection should be performed (Table 2, Fig. 2). In a systematic review of over 1725 cases of TIO, the TIO‐causing tumors were identified in 1493 patients, and surgery was successful in >90% of these patients [40]. However, the difficulty of early recognition and diagnosis of TIO has been demonstrated by a review of 252 patients with TIO, for whom the average time from onset of symptoms to surgical removal of tumor was 5.4 (standard deviation 4.2) years [30]. The location of the tumor will determine the surgical specialist best qualified to perform the resection [89]. Because a complete removal of the tumor is curative, in collaboration with the surgeon, a wide resection with negative tumor margins should be planned.

### Nonsurgical ablation

Radiofrequency ablation [69, 90] or cryoablation [91, 92] have been used to treat TIO when the location of a tumor has made it difficult to completely resect (Fig. 2), although the success rate is variable [92] and long‐term outcomes are unknown.

## Patient follow‐up and monitoring in collaboration with specialists

### Complete resection

Following successful surgery, biochemical abnormalities rapidly reverse: Intact FGF23 levels typically return to normal levels within 24 h, whereas C‐terminal FGF23 levels may take several days to normalize [35, 64, 80, 93, 94]. Patients may experience hungry bone syndrome (HBS) [43, 95, 96] as bones are being remineralized, characterized by secondary hyperparathyroidism, with or without hypocalcemia, hypophosphatemia, and/or hypomagnesemia. The recommended treatment for HBS includes calcium and vitamin D supplementation [97, 98]. There is a rapid, marked, but transient increase in serum 1,25(OH)_2_D following successful resection [80]. Serum phosphate normalization is usually observed within 5 days post‐surgery [1, 80]. Osteomalacia and ALP levels may take ≤1 year to recover [1, 3], although certain skeletal deformities will be irreversible [33]. Serum phosphate should be measured frequently until normalization and then annually (Fig. 2).

### Unresectable, persistent, or recurrent tumors

Tumor recurrence has been reported ≤20 years after initial resection [99]. In a recent Italian study, all patients undergoing lesion biopsy before surgery had tumor recurrence [100]. Another Italian case series identified 16 patients who experienced persistent/recurrent TIO or were diagnosed with TIO in the absence of tumor localization, which was 54.2% (±36.2%) of all identified patients with TIO in the study [24]. In persistent and/or recurrent TIO, the diagnostic approach—including the biochemical evaluation, and functional and anatomical imaging—should be repeated to detect metastasis or multiple foci and to exclude other diseases (Fig. 2). Recurrent and multifocal tumors should be localized and resected if possible [22].

Some patients have persistent hypophosphatemia and elevated FGF23 levels after resection and need to be monitored. If 1,25(OH)_2_D does not rebound quickly, this could indicate residual disease [35, 64]. The need for ongoing treatment and/or continued monitoring highlights the importance of collaboration between referring clinicians and specialists [35, 93].

### Medical therapy for TIO

Therapy should be initiated as soon as a biochemical diagnosis has been made to minimize HBS and prepare the skeleton for surgery (Table 2, Fig. 2). In patients with nonlocalizable, nonresectable, or recurrent tumors, the therapeutic goal of medical treatment is to ease clinical symptoms, restore phosphate homeostasis, and normalize ALP and PTH levels [22, 48, 101].

Traditionally, the approach to medical therapy has been conventional therapy: phosphate salts and active vitamin D (calcitriol, alfacalcidol) [7, 101, 102, 103]. However, these have a narrow therapeutic range, require multiple daily doses and intensive monitoring, and are often difficult to tolerate. Side effects include gastrointestinal intolerance, nephrolithiasis, nephrocalcinosis, impaired renal function, and secondary/tertiary hyperparathyroidism [28, 101]. It is important not to treat with phosphate salts alone due to their tendency to induce hyperparathyroidism and their limited effectiveness.

Cinacalcet is a calcimimetic that decreases PTH, specifically demonstrated in a phase 3 study in patients with primary hyperparathyroidism [104], and increases phosphate reabsorption, which has demonstrated some benefits. However, it can result in hypocalcemia and compound the hypercalciuria that can occur with calcitriol therapy, requiring monitoring of urinary calcium and, in some cases, the addition of thiazide diuretics [102, 105]. Cinacalcet requires further study in TIO.

Burosumab, a human monoclonal antibody against FGF23 that blocks the action of FGF23, offers a promising new therapeutic option. It is approved for the treatment of nonresectable/nonlocalizable TIO in the EU, USA, Japan, and China. Two phase 2 studies have demonstrated that burosumab restores phosphate homeostasis and improves osteomalacia, fracture healing, bone pain, functional mobility, and health‐related QoL for patients with TIO [106, 107]. However, neither burosumab nor phosphate salts with active vitamin D halt tumor progression. Although further long‐term data are required, individual case reports have indicated good efficacy and an acceptable safety profile in one patient treated with burosumab for 6 months [108] and in another for >2 years [109]. In addition, an initial case report indicated that starting burosumab ahead of surgery may be of use in reducing the post‐surgery effects of HBS and hyperparathyroidism, though more research would be required to verify this approach [96]. It should be noted that, because currently available assays are unable to differentiate between burosumab‐bound and free FGF23 [58, 110], FGF23 levels cannot be interpreted in patients treated with burosumab. Although there are no reports directly comparing effects and safety of burosumab with conventional therapy in patients with TIO, the abovementioned results together with a report of a phase 3 study in children with XLH [101] suggest that burosumab is preferable to conventional therapy.

Use of infigratinib, an FGF receptor 1–3 inhibitor, was being investigated as a treatment for unresectable or nonlocalized PMTs, but the clinical trial was terminated due to a greater than expected incidence of ocular adverse events and for not meeting the primary endpoint (NCT03510455) [111]. In silico research is also ongoing to identify small‐molecule inhibitors of FGF23 using hot spot prediction and molecular docking to α‐Klotho [112], which has the potential to lead to the development of further new treatments for TIO.

Treatment with conventional therapy using active vitamin D and phosphate is a reasonable initial approach during the initial search for a tumor and while awaiting surgical resection, in order to heal the osteomalacia. In the setting of severe hypophosphatemic symptoms, unresectable tumor, or a presumed TIO with a nonlocalizable tumor requiring long‐term treatment, burosumab may be advantageous to provide symptomatic improvement and better biochemical control and healing of osteomalacia, using the protocol described by Jan de Beur et al. [107].

### Patient QoL

Patient QoL is an important clinical outcome to consider, although few studies have quantified QoL in patients with TIO [37]. Despite a lack of specific data pertaining to patients with TIO, QoL can be compromised by skeletal deformities or fractures, muscle weakness, fatigue, and bone pain, which can also restrict movement. The impact of chronic pain in general on patients’ QoL has been appreciated for some time [113, 114]. Reduced QoL in patients experiencing chronic pain compared with both the general population and with patients with other chronic conditions has been reported, with several domains of QoL affected, including physical function, interference with professional, family, and social life, and interference with mood [114]. One study in patients with TIO found higher levels of fatigue and lower values in the physical domains of the 36‐item Short Form Survey than in reference populations and severe pain‐based interference with everyday activities [115]. Another phase 2 study indicated that burosumab treatment significantly reduced pain severity and interference, reduced global fatigue, and improved physical mobility [107].

Although it is not always possible to use validated patient‐reported outcome measures or other quantitative measures specific to the patient burden of TIO, it is important to assess the impact of TIO on physical functionality, social relationships, and mental well‐being in these patients [3].

## Summary

TIO has a lengthy diagnostic pathway, with patients’ first point of contact often being a primary care physician. Early recognition of TIO and appropriate referral to specialists are of vital importance to help prevent lifelong deficits and physical deformities [33].

Recognition and diagnosis are obfuscated by the nonspecific symptoms of TIO [3, 64]. This frequently leads to evaluation by physicians who may not have expertise or experience with this rare condition, resulting in delays in the diagnosis and treatment of TIO.

The key factor for improving identification and diagnosis of TIO is to raise awareness among physicians that serum phosphate should be measured in all patients with progressive weakness, unexplained bone and muscle pain, sudden onset of bone stress, or pseudofractures. However, serum phosphate measurement is not typically included in standard chemistry panels [1], so the clinician must think to order this test specifically.

Regional availability and variations relating to diagnostic tests, referral pathways, and imaging modalities may contribute to global inconsistencies in TIO management. With the development of this document, which provides global guidance, we are unable to specifically address all country‐dependent considerations concerning testing, referral, and diagnosis but hope to provide direction relating to clinical red flags, appropriate tests to consider, when to refer for treatment and to whom.

In order to continue improving TIO management in the future, there are several needs: (1) a clear algorithm for initial biochemical testing to support clinical suspicion; (2) better functional imaging; (3) clinically available testing that can differentiate patients with TIO from those with genetic causes—currently, negative genetic test results still do not completely rule out the possibility of genetic diseases; (4) development of improved surgical and other less invasive techniques, such as cryotherapy, radiofrequency therapy, and radiotherapy with somatostatin analogues, to achieve cure even when tumors are located near vital structures and/or remote locations; (5) direct comparison of the efficacy and safety of conventional therapy versus burosumab; (6) increased understanding of FGF23 levels in TIO and other hypophosphatemic disorders, in order to correctly diagnose TIO patients with inappropriately normal levels of FGF23 [116, 117]; (7) more understanding of the *FN1‐FGFR1* fusion gene effects in the pathogenesis of TIO and how to exploit it as a potential therapeutic target; (8) better understanding of severe myopathy seen in patients with TIO; and (9) increased understanding of the recurrent and malignant transformation of PMT.

As a rare disease, there is currently a paucity of high‐quality evidence (e.g., randomized clinical trials) on TIO to aid understanding of the nature of the disease and its burden on patients. In line with our own observations, the authors of a recent literature review on the burden of TIO on patients also commented that most published evidence currently available for TIO is in the form of case studies or case series [37]. This underscores the need for further, well‐designed studies to better characterize signs and symptoms of TIO along with the burden of disease on patients. In the absence of robust data, we aim to support physicians to make the most appropriate decisions in their everyday practice when presented with patients suspected of having TIO, by providing practical recommendations and giving expert‐led opinions. Increased understanding of TIO and standardization of the patient pathway, along with advances in diagnostic assays and treatment modalities, should lead to improved patient outcomes in the future [3, 22, 34, 106, 107, 118, 119].

## Conflicts of interest

Suzanne M. Jan de Beur has received grants from Ultragenyx, Mereo BioPharma (paid to a clinical trial site on which she was the principal investigator) personal fees from Ultragenyx, Amgen, Inozyme Pharma, fees from Ascendis Pharma for serving on advisory boards, and honoraria for lectures from Ultragenyx; she is on the scientific advisory board of the XLH Network Patient Advocacy Group and is past president of the American Society of Bone and Mineral Research. Salvatore Minisola has received royalties or licenses from Abiogen, Kyowa Kirin, Pfizer, and UCB, and payment or honoraria from Amgen, Bruno Farmaceutici, DiaSorin, Eli Lilly, Kyowa Kirin, Sandoz, and Takeda. Wei‐bo Xia has no conflicts of interest to declare. Bo Abrahamsen has received grants from Kyowa Kirin, Novartis, Pharmacosmos, and UCB, speaker fees from Amgen, Eli Lilly, and Pharmacosmos, and consulting fees from Kyowa Kirin and UCB. Jean‐Jacques Body has received consulting and/or speaker fees from Alexion, Amgen, Sandoz, and UCB. Maria Luisa Brandi has received honoraria from Amgen, Bruno Farmaceutici S.p.A., Calcilytix, Kyowa Kirin, and UCB, grants or speaker fees from Abiogen Pharma, Alexion, Amgen, Bruno Farmaceutici S.p.A., Echolight, Eli Lilly and Company, Kyowa Kirin, S.p.A., Theramex, and UCB, and consulting fees from Aboca, Alexion, Amolyt Pharma, Bruno Farmaceutici S.p.A., Calcilytix, Kyowa Kirin, and UCB. Roderick Clifton‐Bligh has received a personal payment from Kyowa Kirin for participation on an advisory board. Michael Collins is supported by the Division of Intramural Research, National Institute of Dental and Craniofacial Research, National Institutes of Health. Pablo Florenzano has received grants from Ultragenyx, consulting fees from Kyowa Kirin, and presentation fees from Ultragenyx. Pascal Houillier has received a grant from Takeda/Sire (payment to an association), consulting fees from Kyowa Kirin and Takeda/Shire, payment or honoraria from Takeda/Shire, and personal fees from Takeda/Shire for advisory boards. Yasuo Imanishi has received payment for lectures from Kyowa Kirin. Erik A. Imel has received grants from Kyowa Kirin and Ultragenyx (paid to his institution) and personal fees for participating in advisory boards and steering committees from Ultragenyx. Aliya A. Khan has received grants and honoraria from Alexion, Amgen, Ascendis, Chugai, Radius, Takeda, and Ultragenyx. M. Carola Zillikens has received grants from Kyowa Kirin and Health Holland (paid to institution), is a member of the steering committee of the European XLH Registry, the steering committee of ERN BOND, the Dutch Guideline for Osteoporosis and Fractures, and is a past board member of the European Calcified Tissue Society. Seiji Fukumoto has received personal fees from Kyowa Kirin and Chugai Pharmaceutical for lectures given and is the President of the Japanese Society for Bone and Mineral Research.

## Author contributions

*Writing—original draft; Writing—review and editing*: Salvatore Minisola, Wei‐bo Xia, Bo Abrahamsen, Jean‐Jacques Body, Maria Luisa Brandi, Roderick Clifton‐Bligh, Michael Collins, Pablo Florenzano, Pascal Houillier, Yasuo Imanishi, Carola Zillikens, Seiji Fukumoto.
